# Incidentally Diagnosed Anomalous Right Coronary Artery with an Interarterial Course Presenting as Chest Pain

**DOI:** 10.7759/cureus.5264

**Published:** 2019-07-29

**Authors:** Kushani Gajjar, Abhas Khurana, Aadhar Patil, Arish Noor, Joyce Meng

**Affiliations:** 1 Cardiology, Allegheny General Hospital, Pittsburgh, USA; 2 Internal Medicine, University of Connecticut, Hartford, USA; 3 Internal Medicine, University of Connecticut, Farmington, USA; 4 Internal Medicine, University of Connecitcut, Hartford, USA; 5 Cardiology, University of Connecticut, Farmington, USA

**Keywords:** anomalous coronary, chest pain, ccta, arca-lcc-ia, malignant inter-arterial arca

## Abstract

The frequency of advanced cardiopulmonary imaging has increased the incidence of diagnosis of coronary artery anomalies, but this poses an interesting management dilemma of what to do with them once these anomalies are found. We present the case of a 57-year-old female with a past medical history of postpartum cardiomyopathy, recovered heart failure with reduced ejection fraction (EF), and alcohol use disorder who presented with chest pain, shortness of breath, nausea, vomiting, and palpitations. A CT angiogram was performed to rule out pulmonary embolism. No pulmonary embolism was found; however, the CT scan revealed an anomalous right coronary artery originating from the left coronary cusp, which had a malignant interarterial course (ARCA-LCC-IA) with a right dominant pattern of myocardial circulation. Subsequent nuclear stress testing did not show evidence of ischemia. Echocardiogram revealed a recurrently reduced EF of 40%. Our patient poses a management dilemma since she presented with possible angina and was found to have an anomalous right coronary artery (ARCA) with a malignant course, but subsequently she had a negative exercise stress test with nuclear perfusion imaging. We will review the literature on ARCA-LCC-IA and its clinical manifestations both generally and with its connection to this case as well as its management. We discuss the incidence, diagnosis, and management of ARCA-LCC-IA, with a focus on incidentally found lesions.

## Introduction

Chest pain as a symptom has a broad differential diagnosis. It is important to consider both the most common and the most concerning causes of chest pain. Coronary artery anomalies (CAAs) can present as chest pain and should be considered in the differential diagnosis of angina if the more common causes are ruled out. The frequency of advanced cardiopulmonary imaging has increased the incidence of diagnosis of CAAs, but this poses an interesting management dilemma of what to do with these anomalies once they are found.

## Case presentation

**Figure 1:**
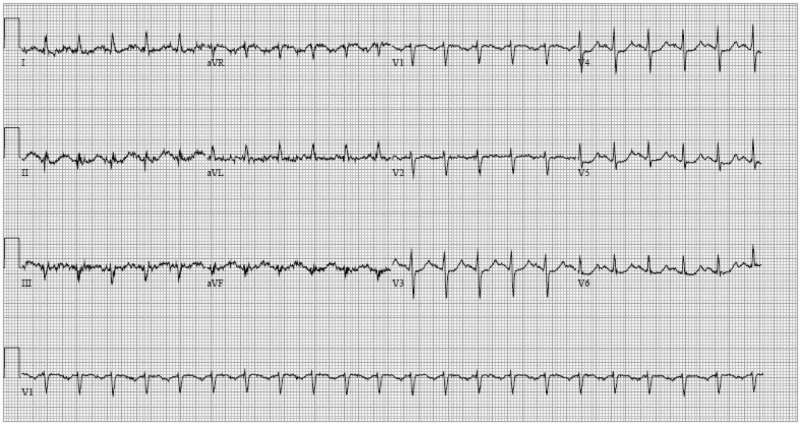
Electrocardiogram (EKG) on Presentation

A 57-year-old female with a past medical history of postpartum cardiomyopathy, recovered heart failure with reduced ejection fraction (HFrEF), alcohol use disorder (denied recent usage), generalized anxiety disorder, and hypothyroidism presented with chest pain, shortness of breath, nausea, vomiting, and palpitations. These symptoms had been going on for three days prior to admission. The chest pain was located in the center of her chest, was pressure-like in quality, radiated to her neck, was 8 on 10 in intensity, and was worse with exertion and relieved by rest. The chest pain was associated with sweating and shortness of breath. Physical examination on admission was unremarkable, except for the patient experiencing generalized distress, tachycardia, and diaphoresis. Electrocardiogram (EKG) was indicative of dynamic ischemic changes including new Q waves in the inferior leads and nonspecific ST-T changes in the precordial leads (Figure 1).

**Figure 2:**
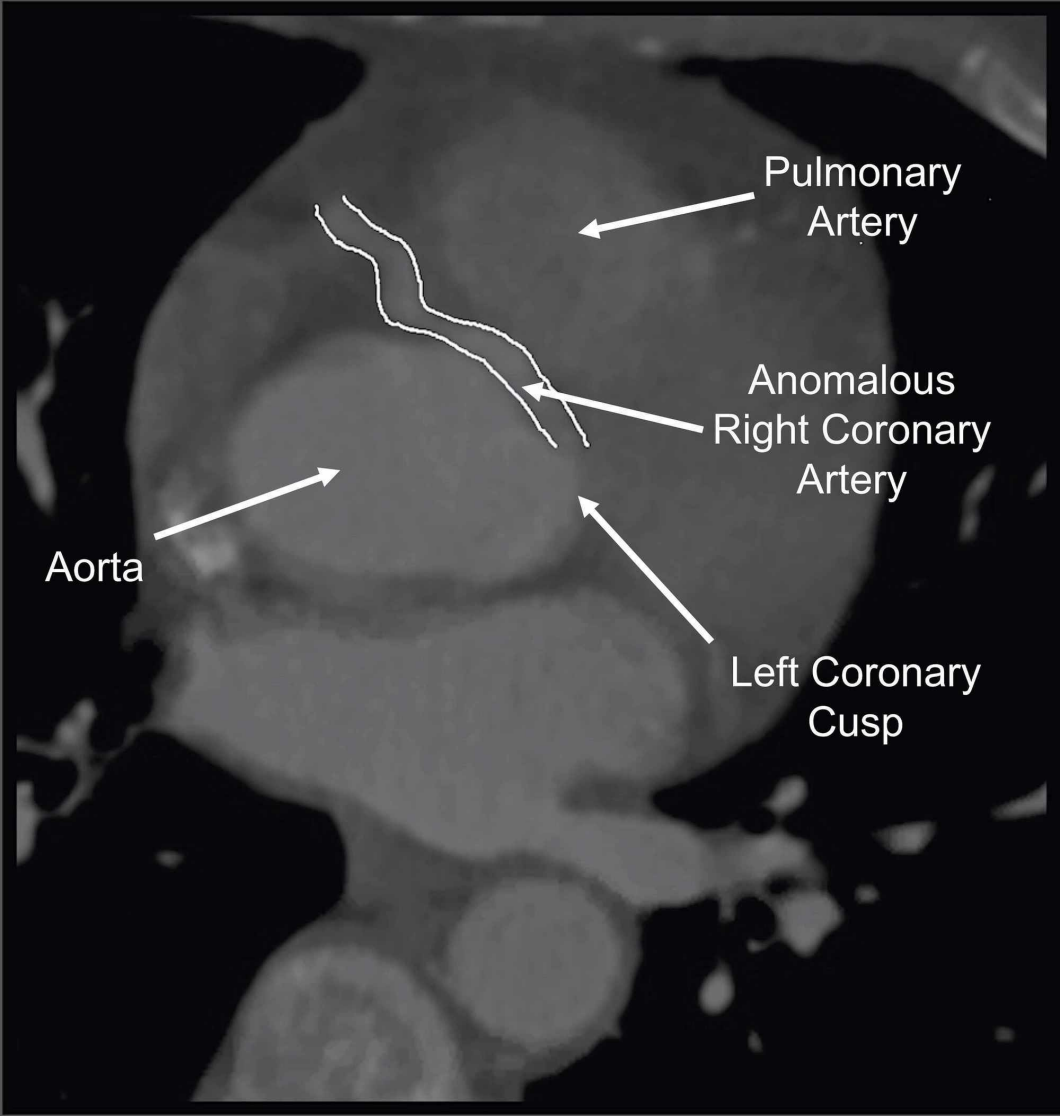
Anomalous Right Coronary Artery Traversing Between the Aorta and Pulmonary Artery in the Patient (Outlined in White)

**Figure 3:**
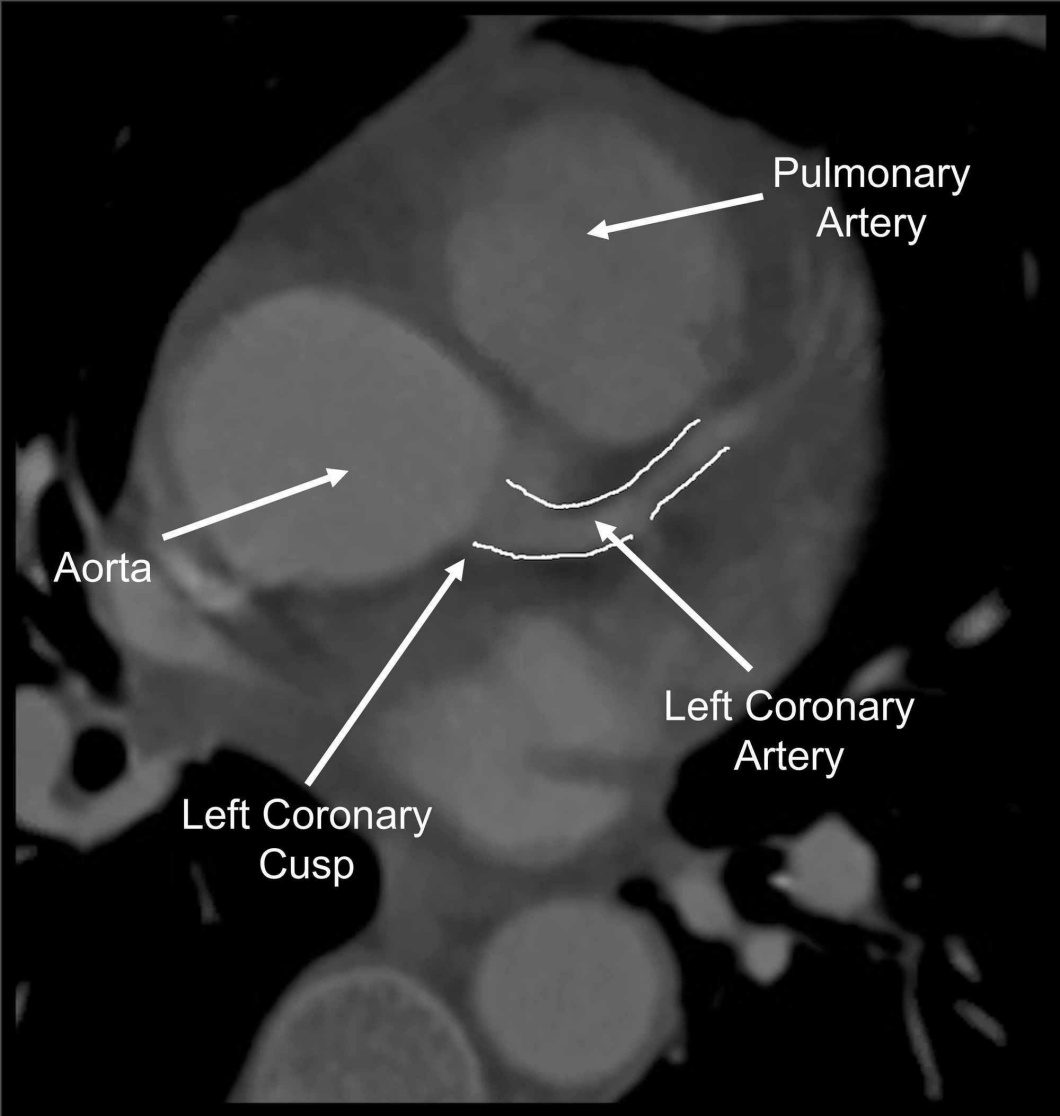
Left Coronary Artery Arising from the Same Left Aortic Cusp As the Right Coronary Artery (Outlined in White)

Three sets of cardiac enzymes drawn six hours apart were within normal limits. CT angiogram (CTA) was performed to rule out pulmonary embolism since the patient’s Wells score for pulmonary embolism was elevated, and she also had a mildly elevated D-dimer of 288 ng/mL. No pulmonary embolism was noted on CTA; however, the study revealed an anomalous right coronary artery (ARCA) originating from the left coronary cusp, which had a malignant interarterial course (ARCA-LCC-IA), with a right dominant pattern of myocardial circulation (Figure 2 and Figure 3).

The patient underwent exercise nuclear stress testing. She was only able to walk 4 minutes and 21 seconds on the treadmill with one+ stages of the Bruce protocol. She did achieve >85% of the maximal predicted heart rate. Perfusion imaging showed no evidence of ischemia. However, transthoracic echocardiogram (TTE) revealed a newly reduced ejection fraction (EF) of 25-35%. Given that the patient had previously been treated for postpartum cardiomyopathy with guideline-directed medical therapy (GDMT) for systolic heart failure resulting in the recovery of EF, she was restarted on GDMT during her hospitalizations. Her symptoms including tachycardia resolved with the reinitiation of aspirin, statin, angiotensin-converting enzyme inhibitor, and beta blocker for HFrEF. The decision was made to not undergo cardiac catheterization as the patient did not have any recurrent symptoms on GDMT and there was no evidence of ischemia on exercise nuclear imaging. The patient continued to follow up with the outpatient cardiology department after discharge. Upon repeat TTE one year after discharge, her EF was found to be 50-55%, with no regional wall abnormalities.

## Discussion

Anomalous aortic origin of the coronary artery is a rare congenital condition in which the coronary artery arises from the opposite sinus of Valsalva. ARCA is a rare entity with incidence thought to be anywhere from 0.17% to 1.2%. ARCA-LCC-IA represents approximately 20% of this subset [1-4].

Currently, it is recommended that the evaluation of individuals who have survived unexplained aborted sudden cardiac death (SCD) or those with unexplained life-threatening arrhythmia, coronary ischemic symptoms, or left ventricular (LV) dysfunction should include assessment of coronary artery origins and course (Class I, level of evidence [LOE] C) [5]. Diagnostic modalities used to detect this condition include coronary CTA (CCTA), magnetic resonance cardiac angiography (MRCA), transesophageal echocardiograms and nuclear stress to categorize the anatomical and physiological abnormalities with this disease entity. Invasive coronary angiography does not allow for a complete understanding of the anomalous anatomy in three dimensions and its dynamic relationship with surrounding structures [6]. CCTA has been studied as a diagnostic modality and it has been suggested that it may be a feasible option to better understand these anomalies [6-8]. MRCA is a diagnostic modality that is becoming increasingly available and enables assessment of both the anatomy and viability of underlying myocardial territories by evaluating for inducible ischemia [7]. CCTA and MRCA are recommended as the initial screening methods in centers with expertise in such imaging by the 2008 American College of Cardiology/American Heart Association guidelines for the management of congenital heart disease in adults [4]. The limitations of these treatment modalities are the cost, limited availability, risk of claustrophobia, risk of renal injury with CCTA and nephrogenic systemic fibrosis with MRCA as well as contraindications with ferromagnetic implants with MRCAs [6-7].

Treatment strategies for these anomalies, once diagnosed, vary from surgical revascularization to medical therapy to minimally invasive stenting, depending on case presentation and experience of a given center in each treatment modality [4,5,8]. Surgical revascularization is the standard of care for ARCA-LCC-IA when there is evidence of ischemia (Class I, LOE B) or when there is evidence of vascular compromise without evidence of ischemia (Class IIa, LOE C) due to the progression of symptoms (e.g., chest pain, syncope) with medical therapy [2,5,8]. Our patient did not meet the Class I or II criteria for surgical repair since her symptoms subsided and EF recovered with GDMT. However, an interarterial course between the aorta and the pulmonary trunk may also be associated with arrhythmia and syncope apart from SCD and ischemia [1-4]. In 80% of autopsies in athletes with SCD and anomalous coronary artery origins, the affected coronary artery coursed between the aorta and the pulmonary artery, as in our patient, and hence represents a high-risk anomaly [1].

Long-term follow-up of patients with CAAs has identified particular anatomical features that carry a higher risk of morbidity and mortality. These features include an interarterial course, a slit-like coronary ostium, ostial flaps, and an initial course within the aortic wall [3].

At this time, there are no guidelines or studies to better understand the optimum management strategies for incidentally found lesions [4]. Multiple studies have shown that multidetector CT scans can accurately detect coronary anomalies [9-10]. Given the increased prevalence of CTA in the workup of chest pain and other nonspecific cardiac and pulmonary symptoms, this type of dilemma will only become more frequent. We hypothesize that patients with incidentally found lesions undergo advanced imaging studies like a CCTA or an MRCA. Exercise and stress impact the conformation of these anatomical anomalies, thereby impacting coronary flow, myocardial perfusion, and subsequently cardiac function [3]. The development of other common cardiovascular diseases such as coronary artery disease and aortic stenosis likely put these patients at a higher risk of developing ischemia than the general population due to their underlying anatomical variation; hence, we suggest they should have regular follow-ups with a cardiologist [4,11].

## Conclusions

Coronary abnormalities are among the most common congenital cardiovascular anomalies, surpassing in prevalence nearly all others combined. Exercise and stress impact the conformation of CAAs, thereby acutely and adversely impacting coronary flow, myocardial perfusion, and subsequently cardiac function. The learning point from this case is the evaluation of individuals who have survived unexplained aborted SCD or with unexplained life-threatening arrhythmia, coronary ischemic symptoms, or LV dysfunction should include assessments of coronary artery origins and course.
